# Primary health care professionals’ experiences with caring for patients with advanced Huntington’s disease: a qualitative study

**DOI:** 10.1186/s12875-024-02408-2

**Published:** 2024-05-07

**Authors:** Marleen R. van Walsem, Emilie I. Howe, Nada Andelic, Jan C. Frich

**Affiliations:** 1 Department of Neurohabilitation, Oslo University Hospital, P.O. Box 4950, Nydalen, 0424 Oslo Norway; 2 Department of Neurology, Oslo University Hospital, P.O. Box 4950, Nydalen, 0424 Oslo Norway; 3 Institute of Health and Society, CHARM - Research Centre of Habilitation and Rehabilitation Models and Services, University of Oslo, P.O. Box 1130, Blindern, 0318 Oslo Norway; 4 Department of Physical Medicine and Rehabilitation, Oslo University Hospital, P.O. Box 4950, Nydalen, 0424 Oslo Norway; 5 Institute of Health and Society, University of Oslo, Box 1130, Blindern, 0318 Oslo Norway; 6 Diakonhjemmet Hospital, Box 23, Vindern, 0319 Oslo Norway

**Keywords:** Care, Advanced huntington’s disease, Health care professionals, Qualitative research

## Abstract

**Background:**

Huntington’s disease (HD) has substantial impact on patients and carers’ lives. Managing patients in the advanced phase of HD may be challenging to primary health care professionals. The aim of this study is to elicit health care professionals’ experiences of managing the challenges with patients with advanced HD in primary health care.

**Methods:**

We did a qualitative study, collecting data from four focus group interviews with 22 primary health care professionals who had experience with caring for patients with HD in Norway. The data were analysed using a qualitative content analysis method, systematic text condensation.

**Results:**

We found that health care professionals who care for patients with HD in primary health care experience challenges related to patients’ behaviour, family members and caregivers, professionals’ individual competency, and the organizational context. They conveyed that successful care and management of patients with advanced HD was dependent on individuals’ competency and “everyday tactics”, well-functioning teams, and leadership and organizational support.

**Conclusion:**

In addition to individual competencies, including being personally suitable for the job, well-functioning primary care teams, and organization support and training is important for health care professionals’ ability to manage patients with advanced HD in primary health care.

## Introduction

Huntington’s disease (HD) is a neurodegenerative disorder, caused by CAG trinucleotide repeat expansion in the huntingtin gene (HTT). HD affects people in the middle of adult life usually between 30 and 50 years of age and is characterized by movement disorders, specifically chorea (dance-like) movements, a variety of mental symptoms such as mood disturbances, irritability and apathy, as well as a decline in cognitive function resulting in dementia [1, 2].

HD has substantial impact on patients and carers’ lives as well as the health and social care systems [3–6]. Patients require complex long-term multidisciplinary treatment and care, particularly in the advanced stages of the disease [7], and specialist clinics often provide multidisciplinary and team-based care [8]. The literature suggests that specialists need to focus on the mental health aspects, and the provision must be flexible and responsive to current needs [9–11]. A recent study [12] shows that patients struggle with increasing dependence, and that they try to cope with the impact and uncertainty of the disease. Caring for HD patients requires specific knowledge and skills, and tailored approaches. Attitude and qualifications of the nursing staff in residential HD care may help patients to focus on preserved abilities and improve the patients’ perceived quality of life [12].

There is variation in how HD clinics and care is organized globally [8]. The health system in Norway is mainly tax-based with 85% of public funding, and it offers universal access to inhabitants according to need [13]. Specialized HD clinics exists in some public hospitals, and specialized rehabilitation programmes exist. A nation-wide network of publicly funded specialised nursing homes have been established [14] for patients with advanced HD. Patients with HD also receive home-based care and in-patient care in regular nursing homes.

We had an interest in identifying what challenges health care professionals in primary healthcare experience in their work with patients with advanced HD. We were also interested in how health care professionals managed and dealt with these challenges. To understand more about practices associated with good clinical care in the community and residential nursing homes, we did a study to elicit health professionals’ experiences of managing the challenges with patients with advanced HD in primary health care.

## Methods

We used a qualitative and explorative approach, collecting data from four focus group interviews [15]. The reason why we found focus groups the most appropriate data collection method was that we anticipated that the sharing experiences in a group would stimulate individuals to share experiences and reflections. We also anticipated that the group discussion would give us an in-depth understanding of the challenges health care professionals experienced and the strategies they used to manage those challenges.

### Recruitment

We recruited participants through an invitation letter sent to individual professionals who were part of a Norwegian national competency network for HD [14]. Eligible participants were healthcare professionals in homebased care, regular residential nursing homes (a facility for the residential care of older people, senior citizens, or disabled people), and specialised residential nursing homes for patients with HD, in the South-East and Middle region of Norway.

### Interview guide and data collection

We developed and used an interview guide consisting of open-ended and probing questions about challenges and ways of handling these in their daily work with caring for patients with advanced HD, such as: Can you reflect on your experiences working with HD? Can you describe a typical situation in which a patient is experienced as “challenging”? Which factors influence your ability to manage the patient well? We used the interview guide in a flexible manner to ensure that the health professionals’ perspectives were in focus. The interview further included examples for some of the questions, to support the discussion when necessary (Table 1).

**Table 1:** Interview guide

• Can you tell us about your workplace?
• Can you reflect on your experiences working with patients with HD?
• Please, tell us about how you usually work with this patient group?
• Do you have any routines to educate new employees? Which? How do you do this?
• Can you describe a typical situation of a patient being «challenging»?
• Which challenges related to behavior or other difficulties have you experienced?
• How do you manage different types of challenges?
• Which conditions do you think influence the possibilities for you to manage your patients in a good manner?
• What are your experiences from collaborating with family members and caregivers to patients with Huntington’s disease?
• Which role do you think family members and caregiver can and should play?
• What do you think are success factors in the management of patients and families?
• Which measures or external support can influence a successful management?
• Patients with HD may have many needs – to what extent do you think these are being met?

We deliberately chose to mix nurses, auxiliary nurses, and social educators (learning disability nurses) in the focus groups interviews, to stimulate reflections from different backgrounds and perspectives. A moderator and an assisting secretary conducted the focus group interviews. The interviews were recorded and transcribed verbatim.

### Analysis

We used systematic text condensation, a method for cross-case thematic analysis of qualitative data [16], to analyse the material. The analysis comprised of four steps: (i) reading all of the material to obtain an overall impression of health care professionals’ experiences with caring for patients with HD in advanced stages; (ii) identifying meaning units, representing specific challenges and strategies applied to manage these challenges, and coding for these; (iii) condensing the contents of code groups and subgroups; and (iv) summarising the meaning from each code group to generalise descriptions and concepts concerning experiences with managing challenges. The first (MvW) and the last (JCF) author did the primary analysis of the data, and all authors contributed to the analysis. The focus of the analysis was to understand more about challenges health care professionals in primary health experienced in their work with patients HD.

## Results

### Participants

We included 22 primary health care professionals who had experience with caring for patients with HD in Norway. Each of the four focus groups consisted of four to seven health professionals and lasted for 90 to 120 min. The interviews were conducted in-person during 2017 at nursing homes the health care professionals were employed or a meeting room for the municipality primary care division. The four focus groups consisted of a mix of nurses, auxiliary nurses, and social educators (learning disability nurses). After four focus group interviews, our impression was that we had a rich data material and that new themes did not emerge in the last interview.

Fourteen of the health professionals worked in specialized nursing homes for HD, while six worked in regular nursing homes, and two worked in home-based services (Table 2). Four participants were male and eighteen were female. Participants ages ranged from 24 to 62 years, with an average age of 42.7 years. On average, participants had 15.6 years of work experience within health care (range 1.5–38 years) and 6 years (range 1.5–19 years) of experience with providing care to patients with HD.

**Table 2:** Characteristics of the participants (*N* = 22) in the four focus groups

Characteristic	No. (%)
Age, years	
20–29 30–39 40–49 50–59 *≥* 60	4 (18) 5 (23) 6 (27) 4 (18) 3 (14)
Gender	
Female Male	18 (82) 4 (18)
Profession	
(Specialist) nurse Auxiliary nurses/assistants Social educator (learning disability nurse)	8 (36) 11 (50) 3 (14)
Primary healthcare setting	
Homebased service Residential nursing home/home care Specialized residential nursing home	2 (9) 6 (27) 14 (64)
Work experience as health professional, years	
0–9 10–19 20–29 30–39	7 (32) 9 (41) 4 (18) 2 (9)

### Qualitative findings

The participants shared reflections on various challenges they experienced and tactics and strategies to manage these. We elaborate on these themes in further detail below. We have chosen to refer to each focus group interview rather than specifics participants in each group, as we consider that group interview as the data source.

### Verbal and physical aggression

Health care professionals reported experiencing a variety of challenging behaviours in patients with HD, including verbal and physical aggression directed towards other residents and professionals. Some participants reported that patients had spat at, hit, and kicked them, and that they had scars on their hands after physical encounters with patients. Yelling and verbal abuse happened regularly. One participant said:

*“I think almost everyone has been beaten … scratched, spat at, and kicked. It’s almost part of the daily routine. Depending on who you care for. I have scars on my hands. A lot of yelling, name-calling. A lot of verbal aggression” (Focus group 1).*

They pointed out that hitting and kicking could be involuntary due to patients’ motor symptoms, and that it could be difficult to judge if inappropriate physical contact was purposeful or not. Obsessive or repetitive behaviour, such as “hang ups” and patients’ continuous urge to smoke, walk around or move around in a wheelchair, could be challenging while caring for patients. They had experienced patients who did not want to eat or to shower, and who denied cleaning when being incontinent, or who wanted to stay in bed all day. A common challenge was that patients did not want to accept assistance because they did not perceive themselves as having an illness. Health care professionals experienced that these behaviours had consequences for patients’ safety and dignity, as illustrated in this quote:

*“Urge to move, and that he walks and walks until he is getting hurt … and we do not have sheltered and large corridors here … professionals get tired out, and sometimes he can be physically abusive.” (Focus group 1)*.

### Difficulties with communication

Health care professionals further experienced behavioural challenges related to difficulties with communicating, and that patients could be frustrated and angry when people did not understand what they tried to express. In addition, they often experienced that patients tried to find other ways to express themselves.

*“But I think aggression often is a result of communication failure, that we do not understand what the patient is trying to tell us, and the only way the patient can communicate is through “firing” us … “give me some new people.” (Focus group 1)*.

### Difficulties with swallowing

Patients could have difficulties with swallowing, eating, and drinking, which required patience and focus during meal situations. These situations could also cause anxiety in professionals, as they feared that patients would choke on food. Additionally, they encountered patients who refused to eat or drink. They often kept trying to get the patient to start eating and drinking again. Such behaviours however, posed both practical and emotional challenges, as expressed by one participant:

*“Many have swallowing difficulties, so it may be difficult to give them food and something to drink. You feel frustration. It may take an hour for a patient to drink 100–200 ml of fluid. You feel a bit down and exhausted. Or, that someone choose not to eat anything at all. For days. Refuses. And you stand there … nothing you can do.” (Focus group 1).*

### Psychological and emotional challenges

Participants could experience psychological and emotional challenges related to patients’ gradual functional decline. Some professionals had worked very closely with patients daily over several years. Inevitably, they developed a close relationship, and they described experiencing that they in part filled a role as substitute family member, becoming “the brother”, “the sister”, “the daughter or son”, or “the parent”. Drawing boundaries and differentiating their role could be challenging emotionally and morally, and could lead to role conflicts, as illustrated in the following quote:

“*You become very fond of them, so it is very difficult the day they are no longer here. It’s a patient group you become unusually closely attached to.” (Focus group 1)*.

### Interactions with family members

Health care professionals reported experiencing challenges in the interaction with patients’ family members. They met family members who were not involved with the patients and those who had difficulty leaving the care to the healthcare professional even if they were exhausted. They also talked about meeting family members who were in the process of developing HD themselves or who had symptoms of HD. One participant commented:

*“But the good thing is that every Christmas he comes over to decorate with lights and … that’s what he can manage to do, so it is ready decorated for Christmas two months ago … and the siblings who are affected have no contact, so you see family members disappearing more and more.” (Focus group 4).*

Working with family members was seen as an integral part of caring for patients, and health professionals required understanding and respect for family members’ preferences for degree of involvement. Participants described becoming a mediator between family members and the patient. They explained how family members tried to avoid anticipating their own future. Consequently, they may have chosen not to be very involved in caring for the affected individual. Other family members could be strongly involved in caring for the patient while they battled knowing that they themselves may become ill with HD and need care in the future. Providing good care to the patients and representing their interests while at the same time being mindful and balancing the needs of the family carers was described as challenging by the healthcare professionals, as illustrated in this quote:

*“Some family members may come and go, but some are very involved! Some may never be here, and some find it difficult, and they cry as soon as they see the ward, and some wish that it is not a ward for patients with Huntington’s disease but a regular nursing home ward. Some feel that it is important that it is a ward dedicated for HD, and not a regular ward. They are as different as the patients, I think.” (Focus group 1).*

### Individual characteristics

Participants underlined the importance of professionals’ individual competency in caring for patients with HD. They described being “personally suitable” to the job, listing individual qualities such as being humble, trustworthy, and knowing your own limitations. One participant said:

*“Those who are personally suitable [for the job] have a great advantage. Because earning trust needs you to be confident. It is not sufficient to just be a nurse or social educator [learning disability nurse].” (Focus group 2).*

In addition, being creative and being able to think and act “outside the box” was associated with being personally suitable for the job, as illustrated in this quote:

*“Once she stood screaming at me. Then I tried to talk to her, but there was no way I could get through. Then I said: I can’t take this no more. Now I will lie down on your bed – and then I laid down. Then, she laughed.” (Focus group 3).*

Informants experienced that lack of individual competency including personal suitability among health care professionals posed challenges when caring for patients with HD.

### Challenges related to the organizational context

Participants reported challenges related to the organizational context and the workings of the health care system, including lack of stable staffing, too short introductory programs, difficult collaboration with other health care professionals, and time restraints. They underlined that patients need continuity and benefit from meeting the same healthcare professionals as much as possible. High turnover rates made it more difficult to build a stable relationship and understand the individual care needs of the patient.

Participants reported that a general educational introduction program for new employees in permanent or temporary positions was insufficient to prepare them for working with patients with advanced HD. For many, the work demanded too much, in terms of being very physically and mentally demanding, in addition to the complexity and variation of the everyday challenges experienced in providing good care to the patients. Additionally, the participants pointed out that it is not necessarily sufficient to build on theoretical knowledge when caring for the patients.

*“Working with patients with HD, you get nothing done before earning trust, and having built a relation. Of course, after two-three training days, you may be able to do everything that can be counted and know routines … but it takes two-three months or up to half a year to be able to do the good work.” (Focus group 2).*

### Coordination with other health professionals

Participants pointed out challenges related the coordination with other health professionals and a lack of competence and knowledge about HD among health care professionals. They often had to educate collaborators in addition to doing their own job. The participants further reported that time restraints and high levels of stress made it challenging to provide the level of care that the patients needed. They experienced this both because of lack of time for practical execution of their tasks as well as to lacking time to communicate sufficiently with each other without the patient. Time constraints were especially challenging when working with patients with HD. Stressed caregivers affected the patients negatively, and further decreased the healthcare professionals’ ability to perform their job. Healthcare professionals said that reactions of the patients were an indicator of their own stress level, as illustrated in the following quote:

*“So, if a health professional walks in and is a bit stressed, because of lack of time or whatever, because we are not able to hide it even though we think we do, she notices at once. It goes straight into her nervous system. So, in a way she is a very good measure of our own level of stress and how you need to force yourself to be calm so that she will notice the calm in you.” (Focus group 2).*

### Individual competence as a resource

Participants underlined that individual competence was key for provision of good care to patients with advanced HD. Participants emphasised the importance of acquiring knowledge about the individual patient to provide good care and to reduce aggressive behaviour in patients. Finding the right consistence of food or liquid so the patient can eat it in, despite difficulties with swallowing, was one example of individual competency. Professionals had to be able to understand what the patients like and do not like, what their current routines are, but also knowledge about their background and past, traumatic experiences, family composition, previous occupation, hobbies, and interests, as illustrated in this quote:

*“There are so many details. Each and every patient have their own thing … their own routines, all right? You have to know that particular pillow needs to be put in that exact position. If you miss out on these things, if you step in from a temp agency, you may be yelled out of the room.“ (Focus group 1).*

Participants pointed out that learning to know your patient takes time and that many patients’ communication abilities are affected limiting the patient themselves to inform about their past and what is important to them. Additionally, their family carers may be around only limited time or entirely absent, restricting the possibilities to obtain specific knowledge about the patients’ past and preferences. Health care professionals said that gathering this knowledge facilitates to build the necessary safety and confidence for the patient, as well as for themselves. It enables them to match patients with health care professionals when providing care, as expressed in the following quote:

*“It is alpha and omega to understand the family situation, to know who she is, for the good and the bad. It is an advantage to know about difficult family relationships … this is an advantage in our work.“ (Focus group 3).*

Participants further described applying a wide variety of “everyday tactics” in handling the challenging situations during their everyday work. One such tactic was being aware of where they positioned themselves when dealing with patients, for example in meal situations, to promote better contact the patient and to avoid being (involuntarily) kicked or slapped by patients. Another tactic professionals used was using wit or humour both with respect to the patients but also among colleagues. They used humour for patients that could understand and had a sense of irony. For example, healthcare professionals may enter the room dancing. For some patients “flirting” worked if a patient shows specific interest in a healthcare professional. To develop a “toolbox” of tactics requires health care professionals to be constantly aware of the individual patient, as well as knowing their patient very well, concerning what did and did not work:

*“We use flirting and smart manoeuvres intentionally. Smile, looking good. If one patient is very fond of breasts, and he does not want to eat, we chose some of us with big breasts to feed him. We talk about it, as we know what the patient likes… if he does not want to shower; we choose to let one particular person do it. It is the same principle. We use the resources we have … we treat them with respect in a way.” (Focus group 1).*

Other strategies they report as helpful in their daily care provision are thinking “out of the box” and thinking in possibilities (instead of limitations) and play on creating special occasions / activities that the patients appreciate extra.

*“I can’t forget the episode; he is almost as tall as me – six and a half feet – and he came towards me with a chair above his head – and I thought: What in the world will I do now? I had worked with aggressive behavior for 15 years, but in this moment, I felt a bit small. I thought: Okay, I must go ahead. Then I walked two steps towards him, and I thought it would be safer. Then he was surprised, and he lowered the chair. He was used to being the tall and scary guy … and after this he never tried to threaten me again” (Focus group 2).*

They observed that on special occasions their patients managed to do much more than expected, surprising both themselves and the healthcare professionals:

*“We think a lot about possibilities. Some may be very affected, but we take them outside to a barbeque on May 17th [Norwegian Constitution Day]. Everyone out! Set the long table! … Everyone comes and they eat sausages even if they usually need it mashed. Very fascinating!“ (Focus group 1).*

### Being a well-functioning team

Besides individual knowledge and competences, participants expressed that managing patients and families with HD was a team task. Several factors were mentioned as important for well-functioning teams: Communicating regularly, being flexible, feeling secure, receiving psychological and social support within the team as well as recognition of this need higher up in the system, in addition to a climate in which members were recognized and experiences and insights were shared irrespective of education or professional training. Among factors health professionals perceived could promote confidence in teams, were to establish care plans, regular meetings with family members to clarify the degree of involvement, gain trust and agree on plans. In some cases when there is no family member involved, their main contacts were the legal representative.

*“We have a formal structure, but we talk a lot. We listen to a stand-in if something has worked. You can’t say: “No, this will not work … because I have a higher education than you” That will not work! Because they have found something that works … let’s try it out”. (Focus group 1).*

Formal routines for information sharing and exchange, such as care plans and regular meetings were important resources. Reporting at the beginning and at the end of a working shift is essential. They often needed to discuss and share experiences on practices regarding the individual patients. They needed to discuss and talk in order to process and to support each other regarding the challenges they are standing in at the end or beginning of a working shift. Still, patients’ needs and what worked could change quickly, so one could not always rely on documented routines. They explained the importance of continuously communicating with other team members about what works and what suddenly did not work, as this often changes quickly, as illustrated in this quote.

*“In usual nursing homes, even documentation and information flow is a challenge. At this place, it is extra challenging. Things change so quickly. We communicate a lot verbally, but to get this information into a care plan … something may be valid only for only a day […] so we learn that the most important thing to do is to communicate, communicate and communicate”. (Focus group 1)*

Participants reported the importance of allowing and helping each other to set boundaries for oneself and others in the team. Taking care of oneself and the team was seen as essential for providing good healthcare to the patients as illustrated by the following citation:

*“… it may sound stupid what I say now, but – if we take care of each other, then we will be better at taking care of the patients. This is basic theory. You have to put the oxygen mask on yourself before putting it on your child.”(Focus group 1).*

Being able to exchange experiences and anecdotes with other professionals in the team was considered important for coping with the loss of a patient, in addition to staying professional in relation to family members.

### Leadership and organizational support

Participants reported that it is essential that leaders establish formal structures and maintain routines that are important to the clinical team. Lack of support and recognition could increase stress levels, which in turn could affect the ability to provide good quality care to their patient. They emphasized the importance of having leaders that understood this need for sharing, not only covering practical aspects of delivering care, but also the need for social and psychological support among team members. Nevertheless, certain situations could go beyond the capability of the team, and required special attention from leaders in the organization, in order to protect health care professionals. Some experiences with patients are extraordinary demanding for the health care professionals especially when the situation endures, causing distress:

*“We adjust to being beaten by the ones we expect to be beaten by … It is much harder to be beaten by someone who has never hit you before … to understand how exhausting it may be over time … they maybe need to receive counseling by a psychologist … not primarily for the patient, but for the professionals.“ (Focus group 1).*

The participants also reported finding ways to improve hiring new people considering the experienced challenges with hiring new people who will stay in the job. They are more likely to recruit people who did not quit after a short period if being explicit about specific challenges with the condition, and emphasizing individual suitability, such as confronting yourself, in addition to educational knowledge and professional competence. They also inform that despite demanding a lot at times, they will receive a lot in return when caring for patients with HD. In other words, they report adapting tactics when recruiting new personnel by preparing them for job during the hiring process, as illustrated by the following citation:

*«They love us too, and we love them. In a job interview, you may say: «You want to learn a lot about yourself? Then you can work with a patient with HD. Because you are challenged in all thinkable ways … so if you can manage patients with HD you can cope with almost anything, I think». (Focus group 1).*

They further reported the importance of adjusting the introductory training program, by prolonging this. New employees need to learn about standard routines, in addition to insights regarding the individual patients. Especially the latter requires time and for them to be able to guide health care professionals through care processes with the patients. Setting aside more time to train new employees also warrants being there to support them as they acquire the scope of the job, which in turn contributes to a higher probability of a newly hired colleague to stay. Training also helps identifying whether a new hire indeed is in the right place or may have found out it is not after all and doesn’t want to continue. This adjustment facilitated hiring the right people for the job and increases continuity. If the introductory training program is not adjusted, there is much less continuity for both patients and the healthcare professionals already working there at the expense of both the patients, the professionals and the system.

## Discussion

### Main findings

We found that health care professionals who care for patients with HD in primary health care experience challenges related to patients’ behaviour, family members and caregivers, professionals’ individual competency, and the organizational context. They conveyed that successful care and management of patients with advanced HD was dependent on individuals’ competency and “everyday tactics”, well-functioning teams, and leadership and organizational support.

### Behavioural challenges

The participants in our study reported behavioural challenges that are typical symptoms of the neurodegenerative process and cognitive decline in HD and have been described previously [1, 2]. Participants described verbal outbursts and physical aggression but emphasized that these were in some cases related to communication difficulties and motor symptoms. They experienced that repetitive behaviour and declining assistance with basic hygiene tasks affected the patients’ safety and dignity, sometimes leading to feelings of exhaustion and moral dilemmas. Health professionals’ account of family members’ and caregivers’ reactions and concerns are in line with previous research [3–5], but clearly represent a challenge in the primary care setting. They talked about varying degree of family involvement, sometimes having to take the role as mediator between patients and family members, and balancing the need to respect and take care of the patient while simultaneously respecting the wishes of family members who in some cases were in the process of developing HD themselves. We found that successful management of these challenges was linked with professionals’ individual competence and suitability, how well the team functioned, and characteristics of the organizational context.

### Individual professionals’ competencies

In addition to formal training and theoretical knowledge, the health professionals underlined the importance of individual professionals’ competencies when trying to understand behavioural challenges and how these can be managed through individually tailored approaches [12, 17]. Being able to think outside the box, taking a flexible approach and knowing your own limitations were viewed as key characteristics. The health professional’s further spoke about the importance of a good “match” between the patient and their carer, and that achieving this required the carer to become acquainted with current routines but also the patient’s background and experiences. We found that being “personally suitable” for the task was seen as an important factor, and that part of individuals’ competency was to be able to use “everyday tactics” individually tailored to the patient at hand. Previous research has highlighted the importance of a safe environment and routines for patients with HD and at the same time being able to adjust approaches to the individual at hand and to provide flexible care is also important when caring for patients with HD [17–19]. Still, the complexity of healthcare in late phase of HD requires integration of knowledge and practices from different professional groups.

### Well-functioning primary care teams

We found that a well-functioning team was seen as a success factor for managing patients with HD in primary health care. Our finding is in line with literature that highlights the role of specialist multi-disciplinary HD expertise in specialist HD care [8, 17]. Teamwork is key in health care [20], but coordination within primary health care may be challenging due to poor information flow and few opportunities for dialogue [21, 22]. In primary health care, health professionals will usually not have immediate access to the multidisciplinary expert competency one may find in a specialists HD clinic but must rely on their individual competency and “the wisdom” of their team. A care plan, which is structured, systematic, and updated is an important resource for primary care teams.

Our findings highlight the importance of teamwork, a learning environment, time to listen and talk, and continuous coordination within the care team [23, 24]. Relational coordination (RC) is a theoretical concept that emphasizes that communication and relationships are crucial in relational interdependent work processes, as in caregiving, underlying more technical tasks [25]. Three essential dimensions of relationships within a team are important for effective coordination: (i) shared knowledge, (ii) shared goals, and (iii) mutual respect for one another’s contributions. Preconditions for high-quality communication in relationships are frequency, timeliness, accuracy and a problem-solving orientation. We think our findings suggests that building and maintaining well-functioning teams is crucial for successful management of patients with HD in primary health care.

### Organizational support and training

Our findings suggest that a systematic organizational approach for onboarding new hires and support from the organization and leaders is important for establishing and maintaining well-functioning teams. This is in line with findings from previous research [26]. In addition, caring for patients with HD in advanced stages requires a lot on the personal level and is demanding both physically and emotionally. A training program for healthcare professionals could promote individuals’ competency and capacity for efficient teamwork. Still, theoretical knowledge about HD or about certain aspects of providing care may not be sufficient. Individuals and teams must gain specific knowledge about the patient both with regards to patients’ background, needs, routines and what works and doesn’t work. Organizational support from leaders that recognize that teams need time to talk and to coordinate efforts could promote better care [24]. Counselling by experts may promote psychological support to members of the team and help the team revise and tailor its approaches to care.

### Methodological considerations

This explorative study was designed to elicit health professionals’ experiences of challenges encountered and managing these challenges with patients with advanced HD in primary health care. The number of participants in the study is limited, and the sample consists of professionals with extensive experience working with patients with HD. The study thus may not highlight experiences of professionals with little experience or those who choose to leave. Still, we think our findings are valid and highlight challenges health professionals in primary health care experience in their work, as well as how they managed and dealt with these challenges. Norway is a country with a publicly financed health care system, including community-based services and care, and practices may differ from other health care contexts. Factors associated with successful care may be transferable to other settings.

## Conclusion

In addition to individual competencies, including being personally suitable for the job, well-functioning primary care teams, and organization support and training is important for health care professionals’ ability to manage patients with advanced HD in primary health care.

## Data Availability

The data is available upon reasonable request.

## Abbreviations

HD: Huntington’s disease
HTT: Huntingtin gene
